# Inherent noise appears as a Lévy walk in fish schools

**DOI:** 10.1038/srep10605

**Published:** 2015-06-03

**Authors:** Hisashi Murakami, Takayuki Niizato, Takenori Tomaru, Yuta Nishiyama, Yukio-Pegio Gunji

**Affiliations:** 1 School of Fundamental Science and Engineering, Waseda University, Shinjuku, Tokyo, 1698050, Japan; 2 Faculty of Engineering, Information and Systems, Tsukuba University, Tsukuba, Ibaraki, 3050006, Japan; 3 Research Institute for Science and Engineering, Waseda University, Shinjuku, Tokyo, 1698050, Japan; 4 Science & Technology Entrepreneurship Laboratory (e-square), Osaka University, Suita, Osaka, 5650871, Japan

## Abstract

Recent experimental and observational data have revealed that the internal structures of collective animal groups are not fixed in time. Rather, individuals can produce noise continuously within their group. These individuals’ movements on the inside of the group, which appear to collapse the global order and information transfer, can enable interactions with various neighbors. In this study, we show that noise generated inherently in a school of ayus (*Plecoglossus altivelis*) is characterized by various power-law behaviors. First, we show that individual fish move faster than Brownian walkers with respect to the center of the mass of the school as a super-diffusive behavior, as seen in starling flocks. Second, we assess neighbor shuffling by measuring the duration of pair-wise contact and find that this distribution obeys the power law. Finally, we show that an individual’s movement in the center of a mass reference frame displays a Lévy walk pattern. Our findings suggest that inherent noise (i.e., movements and changes in the relations between neighbors in a directed group) is dynamically self-organized in both time and space. In particular, Lévy walk in schools can be regarded as a well-balanced movement to facilitate dynamic collective motion and information transfer throughout the group.

Although only local interactions are involved, collective animals (e.g., bird flocks and fish schools) exhibit rapidly synchronized movements, appearing to behave as if they were part of a single organism^1^. Information transfer through the entire group when it is exposed to predation is one of the most intriguing aspects of collective animal groups and has been observed, for example, as the propagation of a density wave within a group^2^. In theoretical studies, numerous simulation models have been proposed to understand the spontaneous emergence of the global order and information transfer in a group based on inter-individual interaction in a bottom-up manner^3^,^4^,^5^,^6^,^7^,^8^,^9^. In most models, the explicit alignment rule, according to which an agent matches its velocity with others in its neighborhood, is assumed, although the latest empirical research suggests that there is no evidence of direct matching of velocity and that global polarization results from interactions other than those that follow the explicit alignment rule^10^.

It is clear, however, that information transfer is propagated by local interactions. Such information transfer can occur even if an individual does not change its relative position with respect to its neighbors, but real animals in a collective group can change its relative position with their neighbors^11^. These individuals’ movements on the inside of the group, which appear to collapse the global order and information transfer, can enable interactions with various neighbors and contribute to dynamic collective behavior.

Indeed, recent advances in image analysis have revealed that the internal structures of collective animal groups, in particular flocks and schools, are not fixed in time^12^,^13^,^14^. On one hand, when looking at an instant in time, individuals in a group can appear to be highly polarized and directed. Moreover, even velocity fluctuations of different individuals are correlated with each other^12^. On the other hand, in the long term, it has been observed that there is a noise generated inherently in collective animal groups, i.e., individuals travel in a directed group and perpetually replace their position with neighbors. Cavagna and others^11^ investigated individual motions on the inside of starling flocks by identifying individual birds’ coordinates temporally and observing them in the center of a mass reference frame. In the center of a mass reference frame, one can obtain individuals’ coordinates with respect to the center of gravity and, hence, their relative movements with respect to the center of gravity. In their empirical research, Cavagna and colleagues estimated how much a bird moved within the group by calculating the mean-square displacement. They found that the mean-square displacement as a function against time was well described by a power-law dependence and that birds showed supper-diffusive behavior, i.e., they moved faster than Brownian walkers with respect to the center of the mass of the flock. Moreover, the researchers revealed that the remaining rate of a certain number of nearest neighbors exponentially decayed with time. In other words, the birds perpetually reshuffled their neighbors.

In the present study, we investigate individuals’ movements on the inside of their group under controlled laboratory conditions (Fig. 1a) using juvenile ayu schools (*Plecoglossus altivelis*) with 10, 20, 30, 40, 50 and 60 individuals. By using video cameras with high temporal resolution (120 frames per second) and image processing software, we obtain identified individuals’ trajectories. Then, we investigate diffusion properties and find that ayu fish also move faster than Brownian walkers with respect to the center of the mass of the school, as seen in starling flocks. Next, we assess neighbors’ shuffling by measuring pair-wise contact duration and find that there is no characteristic time scale in which individuals remain neighbors. We show that the individuals’ movements within the group reveal a Lévy walk pattern with a truncated power-law distribution of step length. Finally, we discuss whether inherent noise in the group^15^,^16^ appearing as Lévy walk is well-balanced movement to facilitate dynamic collective motion and information transfer over the whole group rather than merely erroneous random motions derived from velocity matching among individuals.

## Results

### Diffusion in the Fish School

**Figure 2:**
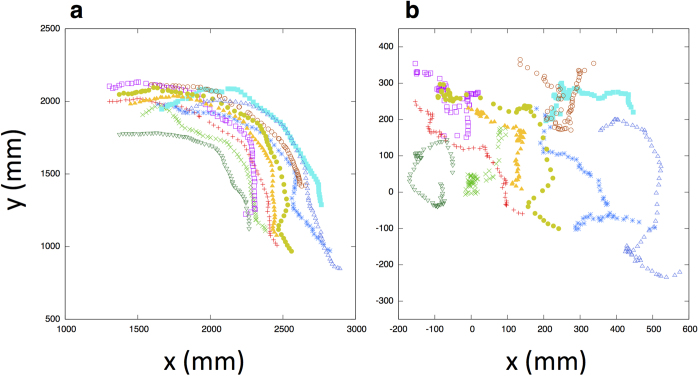
2D reconstruction of some trajectories of different fish in the school. (**a**) Laboratory reference frame. (**b**) Center of mass reference frame. All the axes are in millimeters.

**Figure 3:**
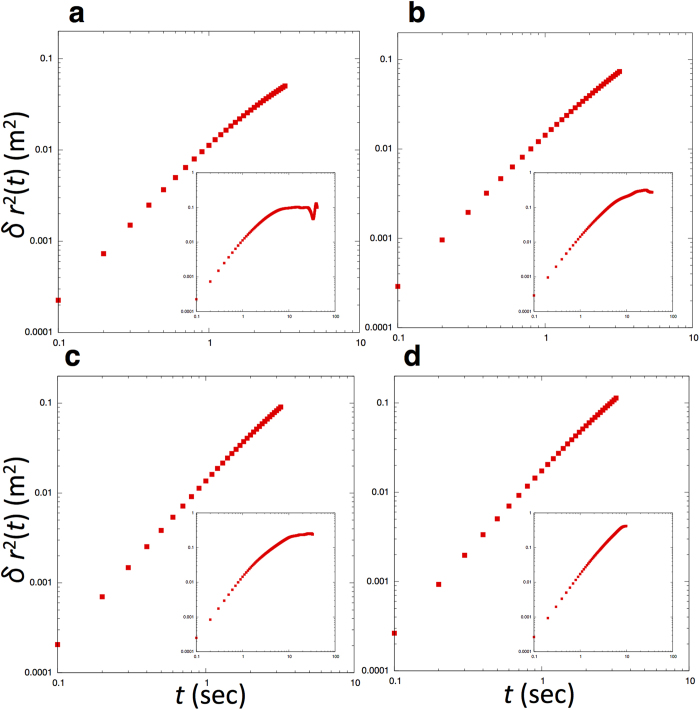
Mean-square displacement in the center of the mass reference frame for four schools of interval [0.1, 3.2]. (**a**) 20 individuals. (**b**) 30 individuals. (**c**) 50 individuals. (**d**) 60 individuals. Insets show the data of the entire time interval.

We first investigated diffusive behavior in the polarized schools, a behavior that has been observed in starling flocks^11^. The center of the mass reference frame is useful for observations of individuals’ movements on the inside of their group. To quantify how much individuals move on the inside of their group, one can use the mean-square displacement in the center of the mass reference frame as a function of time (i.e., at the average amount of distance travelled in a time *t*):
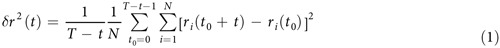
where *R*_*i*_(*t*) indicates the position of *i* at time *t*, *R*_*CM*_(*t*) indicates the position of the center of the mass of the school at time *t*, and *r*_*i*_(*t*) = *R*_*i*_(*t*) − *R*_*CM*_(*t*) therefore represents the position of fish *i* in the center of the mass reference frame. We averaged over all *N* fish and over all time lags of duration *t* in the interval [0, *T*], where *T* is the total time interval. In Fig. 2, we show some trajectories of different fish in the school in the camera’s reference frame and in the center of the mass reference frame. We estimated δ*r*^2^(*t*) with 1.5 orders of magnitude for time (this value is longer than the duration observed in starling flocks^11^), i.e., from 0.1 to 3.2 seconds (see Fig. 3). This approach was taken because although we can estimate how much individuals move by calculating the mean-square displacement in the center of the mass reference frame, the area where fish can move is, of course, restricted in the interior of school. In other words, after the value of the mean-square displacement goes as far as it can go, the value will be restricted due to the border of the group. Therefore, time interval should be restricted as performed in Ref.^11^.

By computing δ*r*^2^(*t*) for schools, we observed that the mean-square displacement in the center of the mass reference was well-described by the following power-law equation:
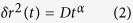
where *α* is the diffusion exponent, falling between 0 and 2, and *D* is the diffusion coefficient. Brownian random walkers diffuse with *α* = 1, corresponding to normal diffusion^17^,^18^. If *α* > 1, walkers or particles show faster diffusion, which is called super-diffusion. If *α* = 2, particles show ballistic diffusion. We found that the diffusion of fish in each school size fits the equation (2) well, with each exponent being larger than 1 (10 individuals: *α* = 1.34, *D* = 0.0054; *N* = 32, *R*^2^ = 0.99, *F* = 41.2, *P* < 10^−18^; 20 individuals: *α* = 1.52, *D* = 0.0099; *N* = 32*, R*^2^ = 0.99, *F* = 186.2, *P* < 10^−28^; 30 individuals: *α* = 1.57, *D* = 0.013; *N* = 32, *R*^2^ = 0.99, *F = *700.7, *P* < 10^−36^; 40 individuals: *α* = 1.63, *D* = 0.017; *N* = 32, *R*^2^ = 0.99, *F* = 3315.7, *P < *10^−47^; 50 individuals: *α* = 1.75, *D* = 0.012; *N* = 32, *R*^2^ = 0.99, *F* = 1084.9, *P* < 10^−39^; 60 individuals: *α* = 1.73, *D* = 0.016; *N* = 32, *R*^2^ = 0.99, *F* = 24543.3, *P* < 10^−60^). Hence, fish display super-diffusive behavior in the center of the mass reference frame. Figure 3 shows the mean-square displacement in the center of the mass reference frame against time for four schools.

### Contact Duration as an Estimation of Neighbor Shuffling

Next, we investigated neighbor shuffling in schools. Cavagna and colleagues^11^ assessed neighbor changing in bird flocks by calculating the proportion of birds that remained as one of a number of nearest neighbors of focal birds, indicating that neighbor changing declined exponentially against time. The authors concluded that neighbor reshuffling does occur and that there was no indication of a preferred structure of neighbors in the flock. Instead of computing such proportions, in this study, we estimate neighbor shuffling by investigating contact duration time.

**Figure 4:**
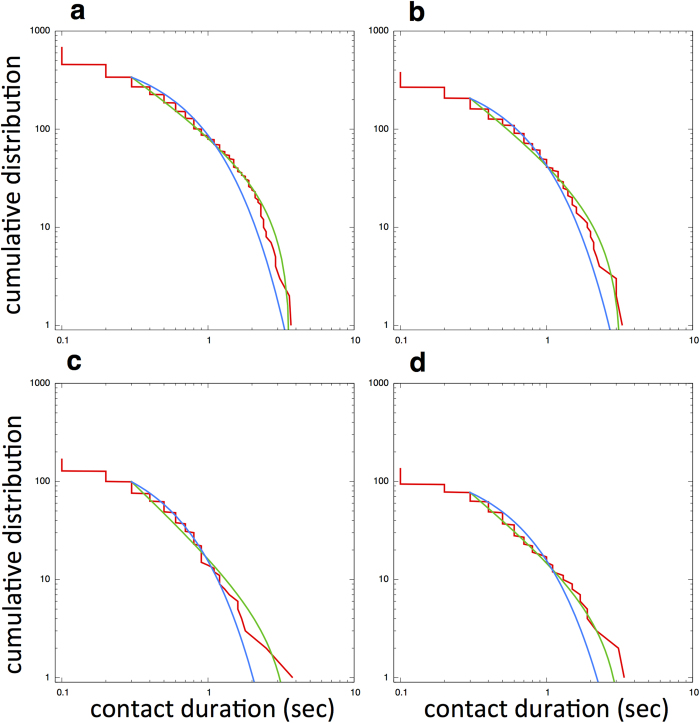
Cumulative distributions of contact duration for four schools. (**a**) Polarized school with 10 individuals. (**b**) Polarized school with 20 individuals. (**c**) Polarized school with 40 individuals. (**d**) Milling school with 40 individuals. The model fits are truncated power-law (green) and exponential (blue) distributions.

Recently, by using mobile devices that can assess mutual proximity in a distributed manner, person-to-person interactions in various human communities (e.g., offices, hospitals, conferences and so on) have been analyzed systematically^19^,^20^,^21^. In such empirical research, it has been revealed that the contact duration time of pairs within a 1-2 m detection range exhibited a power-law distribution. Because this type of analysis indicates how long individuals interact with their neighbors, we can apply it to fish schools to quantify neighbor shuffling using individuals’ temporal coordinates. When we define two fish as a pair if they are within the detection range *r*_*d*_ = 60 (mm), we find that the contact duration of pairs of fish shows a truncated power-law distribution in some polarized schools. Interestingly, the truncated power-law distribution is also found in the milling school (for the statistical values see supplemental information, table S1). These results indicate that neighbor shuffling estimated by contact duration in both the polarized and milling schools has a similar structure in time, which agrees with results observed in human communities. Figure 4 shows the cumulative distribution of the contact duration of three polarized schools and one milling school.

### Lévy Walk in Fish Schools

The super-diffusive movement described above is one of the characteristics of the Lévy walk^18^. The Lévy walk describes a pattern composed of small-step clusters separated by longer relocations^22^, in which the distribution of step-length *l* is as follows:
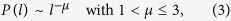
where *μ* represents the power-law exponent. Because animal movements are constrained under various conditions (e.g., due to physiology), the truncated power law is generally thought to better represent movement patterns in nature^23^,^24^. Here, we show that fish behave as Lévy walkers on the inside of schools with the truncated power-law distribution of step lengths.

**Figure 5:**
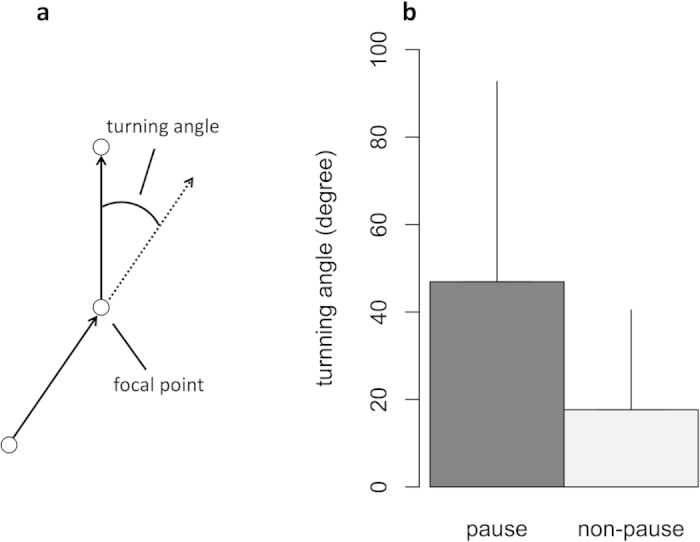
Turning angle at pausing points and non-pausing points. (**a**) Schematic diagram of turning angle. (**b**) Mean turning angle at pausing points (dark grey) and non-pausing points (pale grey). Error bars are SDs.

**Figure 6:**
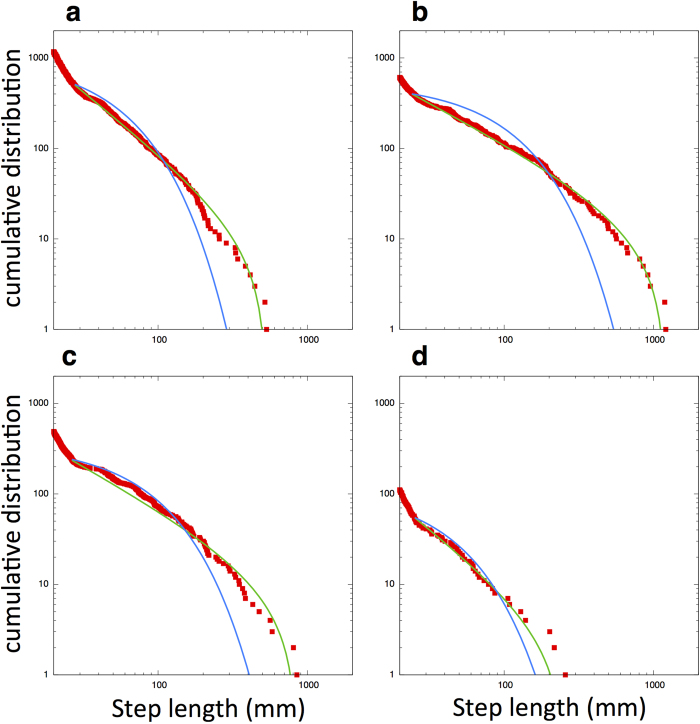
Cumulative distributions of step length in the center of the mass reference frame. (**a**) 10 individuals. (**b**) 40 individuals. (**c**) 60 individuals. (**d**) One individual. The model fits are truncated power-law (green) and exponential (blue) distributions.

**Figure 7:**
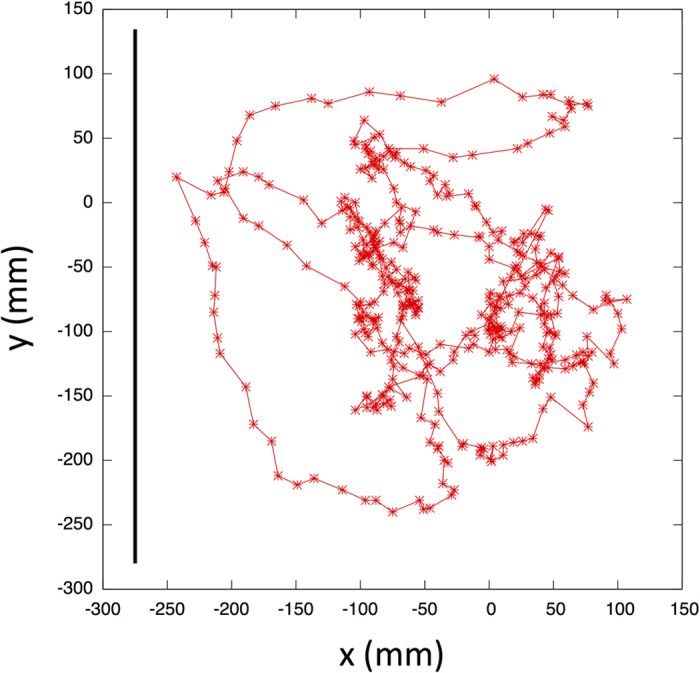
Longer trajectories of an individual in the center of the reference frame. Scale bar represents school size, i.e., mean maximum distance between two individuals shown in Table 1. The axes are in millimeters.

The step length can be defined in different ways, such as the distance between consecutive landings on the sea surface for albatross^25^,^26^,^27^ and the saccade interval length for fruit flies^28^. To investigate whether the fish log-scale movement lengths in the schools followed power-law distributions, we define the step-length as the intermittent interval length^29^ in the trajectories in the center of the mass reference frame as follows: If *dr* < |*r*_*i*_(*t*)−*r*_*i*_(*t*−*dt*)|, i.e., if the distance between consecutive positions of *i* in the center of the mass reference are closer than *dr*, *r*_*i*_(*t*) is considered to be a pausing point. Here, we set *dr* = 20(mm). Note that we also observed the same results of following analysis at around *dt* = 20 (for the case of *dr* = 15 and 25, see supplementary information, figure S1 and table S2). When we calculate turning angle per *dt* at both pausing and non-pausing points for all polarized schools (Fig. 5a), we find that turning angle at pausing points is significantly larger than that at non-pausing points (T-test; pausing points (*N* = 4156, Mean ± SD = 46.91 ± 45.79 (degree)) vs non-pausing points (*N* = 3341, Mean ± SD = 17.63 ± 22.82 (degree)); *p* < 10^−232^, T-value = 76.18) (Fig. 5b), where non-pausing point is defined as a position that is not pausing point and whose one next and one before positions are also not pausing point. The step-length *l* (>*dr*) is then considered as the distance between any two successive pausing points. We find that the step lengths of each polarized school follow a truncated power-law distribution with the exponent *μ* ranging from 1 < *μ* ≤ 3 (for the statistical values see the supplemental information, table S3). Because each exponent *μ* systematically ranges in the interval 1 < *μ* ≤ 3, these results indicate that fish behave as Lévy walkers on the inside of schools. Moreover, when we check an individual’s trajectory, its step lengths also show a truncated power-law distribution (for the distributions of step lengths and the statistical values see the supplemental information, figure S2 and table S4). Figure 6 shows the cumulative distributions of the step lengths of three polarized schools and of an individual. In Fig. 7, we present a longer trajectory of an individual in the center of the mass reference frame (for four more samples see supplementary information, figure S3). Scale bar in these figures indicates the school sizes, i.e., mean maximum distance between individual shown in Table 1. It is easy to see that individuals visit the spatial locations throughout the group, but not stays in a local region within the group, and that there are step clusters separated by longer relocations, which is a characteristic of the trajectory described by a Lévy walk^22^.

## Discussion

We conducted three investigations on the inherent noise in schools of ayus that show polarized and milling patterns, obtaining individual temporal coordinates. First, we calculated the mean-square displacement in the center of the mass reference frame, as seen in starling flocks, and observed that there were super-diffusive behaviors in polarized schools with exponents of *α* > 1. This result indicates that fish in schools diffuse faster than Brownian motion. Although model simulations^30^ have predicted two-dimensional super-diffusion with an exponent of *α* = 4/3, diffusion in real schools occurs faster than predicted, except for schools with 10 individuals. Moreover, we observed the trend that the larger the school size is, the higher the diffusion exponent will be. This relation may be caused by an effect of the area in which the fish can travel, i.e., on the domain covered by a school. If a fish moves beyond the domain, it would be separated from its school. We might, therefore, consider that the smaller the school size is, the more constraints there will be on an individual’s diffusion and that the exponent would reach a certain value in larger schools. Note that schooling fish inevitably contact with the boundary of the tank. Although it seems that mean velocity does not change due to the school size (Table 1), the larger the size is, the larger the boundary effect might become. Therefore, there is also a possibility that the diffusive exponent changes due to the boundary effect.

The polarized and milling patterns are known as emergent collective ordered states in fish schools, which co-exist for the same individual behaviors^10^,^31^. One can discriminate these two self-organized patterns by using two order parameters: polarization parameters and rotation parameters. This condition raises the question as to what type of properties would be commonly observed in both polarized and milling states. For these two states, we calculated pair-wise contact duration, which allows us to quantify neighbor shuffling because it measures how long individuals interact with neighbors. We found that the distributions of contact durations in polarized schools showed a power-law behavior, as have been observed in various human communities. This result indicates the absence of a characteristic scale with respect to how long individuals interact with neighbors. Similarly, we observed the distribution with a power-law behavior in contact duration in the milling school. Therefore, this property of neighbor changing with respect to time is considered to be common both in polarized and milling patterns in fish schools. Note that the way we quantified neighbor shuffling here is different from the method employed by Cavagna and others for starling flocks. They defined neighbors as a number of individuals nearest to a focal individual, whereas we defined neighbors as individuals in the neighborhood of the focal individual within detection range *r*_*d*_. In other words, whereas Cavagna and others used topological neighborhoods in their studies, we used metric neighborhoods to estimate neighbor shuffling^32^.

It seems that if individuals behave ideally, there is no inherent noise and hence no position changing in the collective group. The results discussed above, however, indicate that individuals exhibit super-diffusive behavior within the group, leaving neighbors with no characteristic time scale. Such inherent noise, which might be expected to be detrimental for collectivity, plays an important role in facilitating interactions with various neighbors and thereby robust collective motion and information transfer. Is there a balance between excessive movement that is detrimental to the maintenance of the group and movement that is too slight to contribute to collectivity?

In considering this question, our results show that fish movement lengths in schools follow a truncated power-law distribution, i.e., a Lévy walk. In a study on foraging strategy, a Lévy walk with the power-law exponent *μ* ranging from 1 < *μ* ≤ 3 was considered to be important in a natural environment in which resources are unpredictably distributed^18^. A Lévy walk with *μ* = 2 indicates optimal searching behavior in such an environment. For the exponent *μ*  ≈ 1, movement patterns are close to ballistic motion. This movement is useful to a foraging animal that is exploration foraging if resources are homogeneously distributed far from an animal’s location. For *μ* > 3, the walk is approximated as Brownian motion. This motion is applicable for exploitation foraging if resources are abundantly distributed near an animal’s location. A Lévy walk with the exponent *μ* ranging from 1 < *μ* ≤ 3, therefore, indicates a foraging pattern that balances exploitation and exploration foraging.

We can paraphrase these explanations regarding individual movements within a group with an analogy. If the step lengths of individuals in the center of the reference frame follow the power-law distribution with the exponent *μ*≈1, they might move with much longer step lengths that might be detrimental to collective motion and information transfer through the group. If *μ* > 3, individuals might stay in a local region within the group, and it would be difficult for individuals’ movements within the group to contribute to dynamic collective behavior. A Lévy walk with exponent μ ranging from 1 < *μ* ≤ 3 was observed in schooling ayus, which can be regarded as a well-balanced movement to facilitate dynamic collective motion and information transfer throughout the group. Moreover, we observed that the exponent *μ* was ranging around two (from 1.86 to 2.33). Although this is the same value found for optimal foraging under certain circumstances^26^, there is no food in a school. What can be the resource? We consider that each individual searches “communication” among other individuals; new communication is explored, and familiar communication among neighbors is exploited. Our discovery sheds light on the underlying causes of Lévy walk that is not only search for foods but also communication.

Note again that our results suggest that (i) even though individuals show cohesive schooling behavior with high polarity, (ii) each individual movement relative to the center of the mass of the group displays Lévy walk pattern. It has been reported that anomalous foraging patterns including Lévy walk can emerge from collective foraging dynamics, such as leader-follower and/or fission-fusion dynamics^33^,^34^. These dynamics may partially explain the Lévy walk within the group especially with respect to (ii) as Lévy walk that emerges from inter-individuals interaction. However, more powerful model of collective behavior must be required to understand both (i) and (ii) at the same time.

## Methods

### Ethics statement

This study was carried out in strict accordance with the recommendations in the Guide for the Care and Use of Laboratory Animals of the National Institutes of Health. The protocol was approved by the Committee on the Ethics of Animal Experiments of the University of Tsukuba (Permit Number: 14-386). All efforts were made to minimize suffering.

### Experimental setup

We studied ayu *Plecoglossus altivelis,* also known as sweetfish, which live throughout Japan and are farmed widely in Japan. Juvenile ayus (approximately 7-14 cm in body length) display typical schooling behavior, though adult ayus tend to show territorial behavior in environments where fish density is low^35^. We purchased juveniles from Tarumiyoushoku (Kasumigaura, Ibaraki, Japan) and housed them in a controlled laboratory. Approximately 150 fish lived in a 0.8 m^3^ tank with continuously filtered and recycled fresh water with a temperature maintained at 16.4 °C, and were fed commercial food pellets. Immediately before each experiment was conducted, randomly chosen fish were separated into each school size and moved to an experimental arena without pre-training.

The experimental arena consisted of a 3 × 3 m white shallow tank. The water depth was approximately 8 cm so that schools would be approximately 2D. The fish were recorded with an overhead gray-scale video camera (Library GE 60; Library Co. Ltd., Tokyo, Japan) at a spatial resolution of 640 × 480 pixels and a temporal resolution of 120 frames per second. Schooling fish exhibit two typical ordered states. The first is a polarized state in which they exhibit a turning movement and in which individuals tend to be highly polarized through the group, and the second is a milling state in which individuals exhibit high polarization locally but the group conducts a rotating movement as a whole. In the school of ayus, both of these states were observed. The polarized and milling patterns can be distinguished by using two order parameters^36^—a polarization parameter *O*_*P*_ = (1/*N*)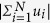 and a rotation parameter *O*_*R*_ = (1/*N*)
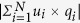
—where *u*_*i*_ is the unit direction of fish number *i* and *q*_*i*_ is the unit vector pointing from the school’s center of mass toward fish *i*. Each order parameter takes values of between 0 (no alignment or rotation) and 1 (strong alignment or rotation). Hence, we defined polarized patterns as those with high values of the polarization parameter and low values of the rotation parameter and milling patterns as those with high values of the rotation parameter and low values of the polarization parameter. In this study, we used 6 schools—with 10, 20, 30, 40, 50 and 60 individuals—for the polarization pattern and one school, with 40 individuals, for the milling pattern for a total of 7 schools (Table 1).

### Tracking

Time series of identified individuals’ positions were tracked using image-processing software (Library Move-tr/2D ver. 8.31; Library Co. Ltd., Tokyo, Japan) on gray-scale images. The shape of each fish and its geometric center were identified by the fact that the fish appear darker than the surrounding area, and the fish trajectories were constructed by tracking individuals from one frame to the next. When fish overlapped or made contact with others, we separated them using the manual tracking mode of the software. As a result, for each observed time duration *T,* we obtained all of the individuals’ x–y coordinates as a single pixel with a side length of 4.76 mm (Table 1). In this study, the time interval between two consecutive reconstructions of individuals’ coordinates was *dt* = 0.1 sec (12 frames).

### Model fitting

In terms of step length and contact duration distributions, we calculated exponents and coefficients of the best-fit model for truncated power laws and exponential models using maximum likelihood estimation and Akaike Information Criteria, and performed a goodness-of-fit procedure, including *G*-test, to determine significance for models^23^,^24^,^25^,^27^,^29^,^37^,^38^,^39^,^40^,^41^. Because animal movements are constrained under various conditions (e.g., due to physiology), the truncated power law is generally thought to better represent movement patterns in nature^24^ given by the following probability density function: *f*(*x*) = (*μ* – 1)/(*x*_*min*_^1–*μ*^ – *x*_*max*_^1–*μ*^)*x*^−*μ*^, where *μ* is the power law exponent, *x*_*min*_ is the start of the tail of the data, and *x*_*max*_ is the maximum value of the data for the model. The probability density function *f*(*x*) of the exponential model is: *f*(*x*) = *λ*exp(−*λ*(*x* − *x*_*min*_)), where *λ* is the exponent for the model. In the case of step lengths, we first determined *x*_*min*_ using Kolmogorov–Smirnov statistics^39^. The *x*_*min*_ was limited equal to or smaller than 30, because large *x*_*min*_ values drastically reduce the effective sample size and narrow the effective range of the data^40^. The *x*_*max*_ was set at the maximum value observed. Second, maximum likelihood estimation was used to obtain best-fit exponents for the truncated power-law and exponential model. Third, log-likelihood was calculated for the truncated power-law and exponential models. Fourth, Akaike Information Criteria and Akaike weight was calculated for both model, and the model with a better support was determined based on Akaike weight. Finally, goodness-of-fit was calculated using *G*-test for the better support model with cumulative distribution functions of truncated power-law. We considered that the model was plausible for the data if the resulting *P* value was greater than 0.1. In the case of contact duration, we simply estimated *x*_*min*_ by eye^41^, because we could not determined *x*_*min*_ of discrete *x* using Kolmogorov–Smirnov statistic. We here estimated two values of *x*_*min*_ at 0.3 and 0.4. Then we considered that the model was plausible for the data if the model was supported by Akaike weight at the both values of *x*_*min*_, and if *P* value of goodness-of-fit was greater than 0.1 at the both values of *x*_*min*_, otherwise neither was plausible. Finally, in the case of step-lengths of individual fish as shown in supplemental information, note that only fish with an effective sample size (i.e., the number of step lengths between *x*_*min*_ and *x*_*max*_) of ≥50 were used in the further analysis, as recommended by Ref. 39.

## Additional Information

**How to cite this article**: Murakami, H. *et al.* Inherent noise appears as a Lévy walk in fish schools. *Sci. Rep.* **5**, 10605; doi: 10.1038/srep10605 (2015).

## Supplementary Material

Supporting Information

## Figures and Tables

**Figure 1:**
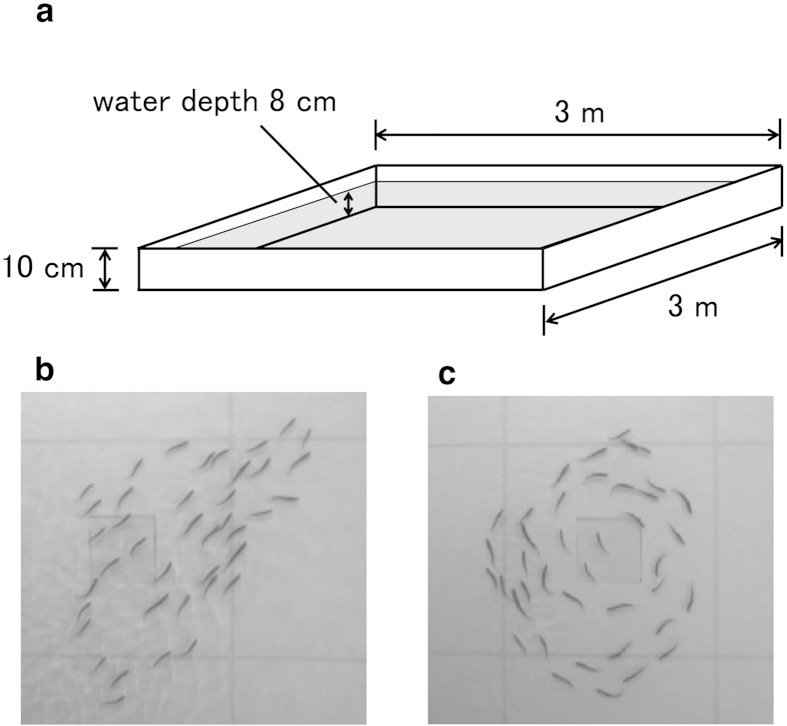
Experimental setup. (**a**) Illustration of experimental tank. The pale grey area represents the water pool. (**b**) A snapshot of polarized school with 40 individuals. (**c**) A snapshot of milling school with 40 individuals.

**Table 1:** Data of analyzed schools. School size is defined as the maximum distance between two fish belonging to the school.

**Number of individuals**	**State of school**	**School size (mm)**	**Velocity (mm/sec)**	**Velocity of the center of the mass (mm/sec)**	**Total time interval (sec)**	***O*_*p*_**	***O*_*r*_**
10	polarized	414.6	234.0	201.2	141.5	0.928	0.111
20	polarized	720.16	217.8	180.2	41.6	0.859	0.183
30	polarized	1232.0	256.5	198.7	36.0	0.888	0.147
40	polarized	1560.3	261.0	207.0	19.1	0.837	0.106
50	polarized	1237.2	212.2	158.9	33.0	0.804	0.105
60	polarized	1557.1	293.7	250.6	10.0	0.922	0.212
40	milling	1557.7	199.5	27.3	52.1	0.139	0.849

*O*_*p*_ and *O*_*r*_ indicate the order parameters for polarization and rotation, respectively. The state of the school is considered as polarized at a high value of *O*_*p*_ and a low value of *O*_*r*_ and as milling at a high value of *O*_*r*_ and a low of *O*_*p*_. See also in the main text.
