# Supplementary material for: Inherent noise appears as a Lévy walk in fish schools
Source: Sci Rep. 2015 Jun 3;5:10605. doi: 10.1038/srep10605 (PMC4454141; doi:10.1038/srep10605)
Supplement: Supporting Information [file srep10605-s1.pdf]

Supplementary Information to "Inherent noise appears as a Lévy walk in fish school"

Hisashi Murakami[1], Takayuki Niizato[2], Takenori Tomaru[1], Yuta Nishiyama[3] and Yukio-Pegio Gunji[4]

[1]Department of Earth & Planetary Sciences, Kobe University, Kobe, Hyogo, Japan
[2]Faculty of Engineering, Information and Systems, Tsukuba University, Tsukuba, Ibaraki, Japan
[3]Science & Technology Entrepreneurship Laboratory (e-square), Osaka University, Suita, Osaka, Japan
[4]School of Fundamental Science and Engineering, Waseda University, Tokyo, Japan

This SI contains supplementary figures S1-S4 and tables S1-S4.

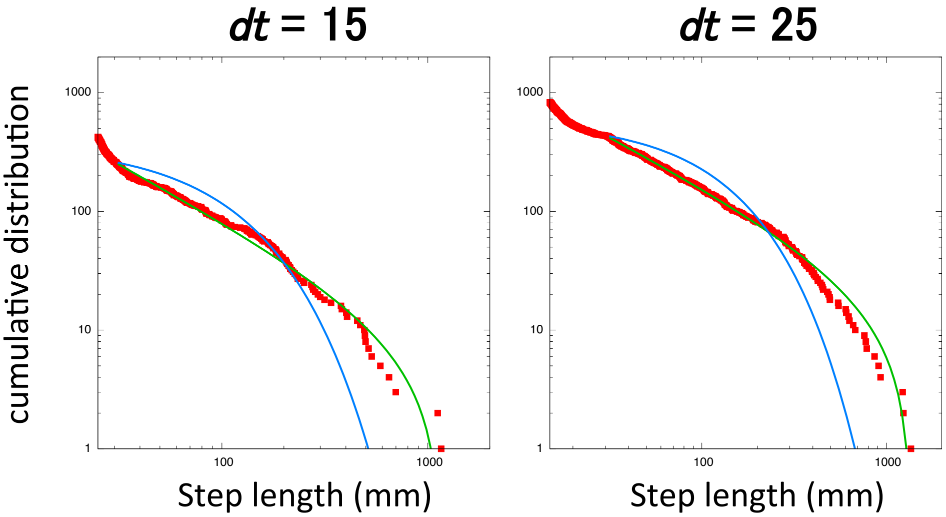

Figure S1. Cumulative distributions of step-lengths in the center of mass reference frame, displayed by 40 individuals school at *dt* = 15 and 25. The model fits are truncated power-law (green) and exponential (blue) distributions. Also see table S1 for the statistical values.

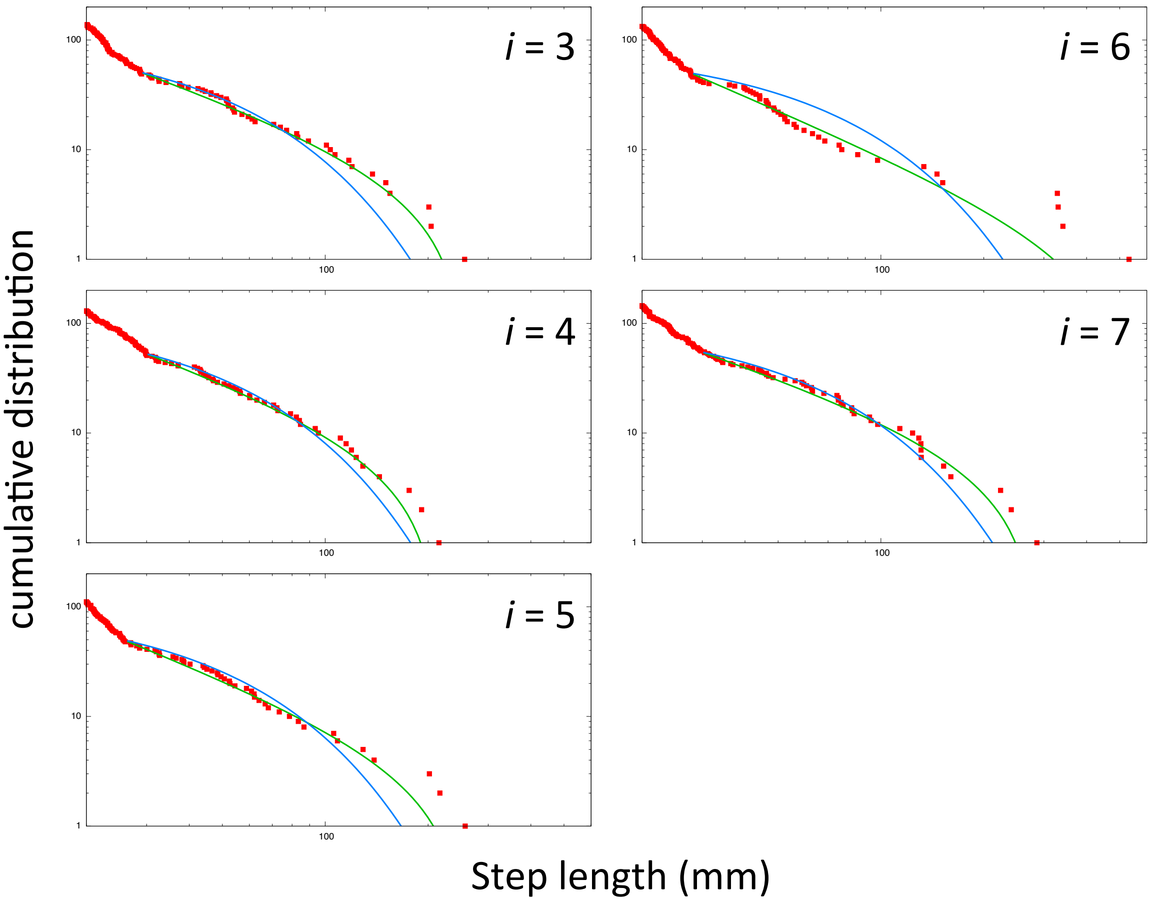

Figure S2. Cumulative distributions of step-lengths in the center of mass reference frame, displayed by individual fish. We here show only fish with an effective sample size (i.e., the number of step lengths between x_{min} and x_{max}) of ≥50. Note that all of them are belonging to school with 10 individuals. Number *i* in each plot indicates individual number of the school that corresponds as in table S4. The model fits are truncated power-law (green) and exponential (blue) distributions.

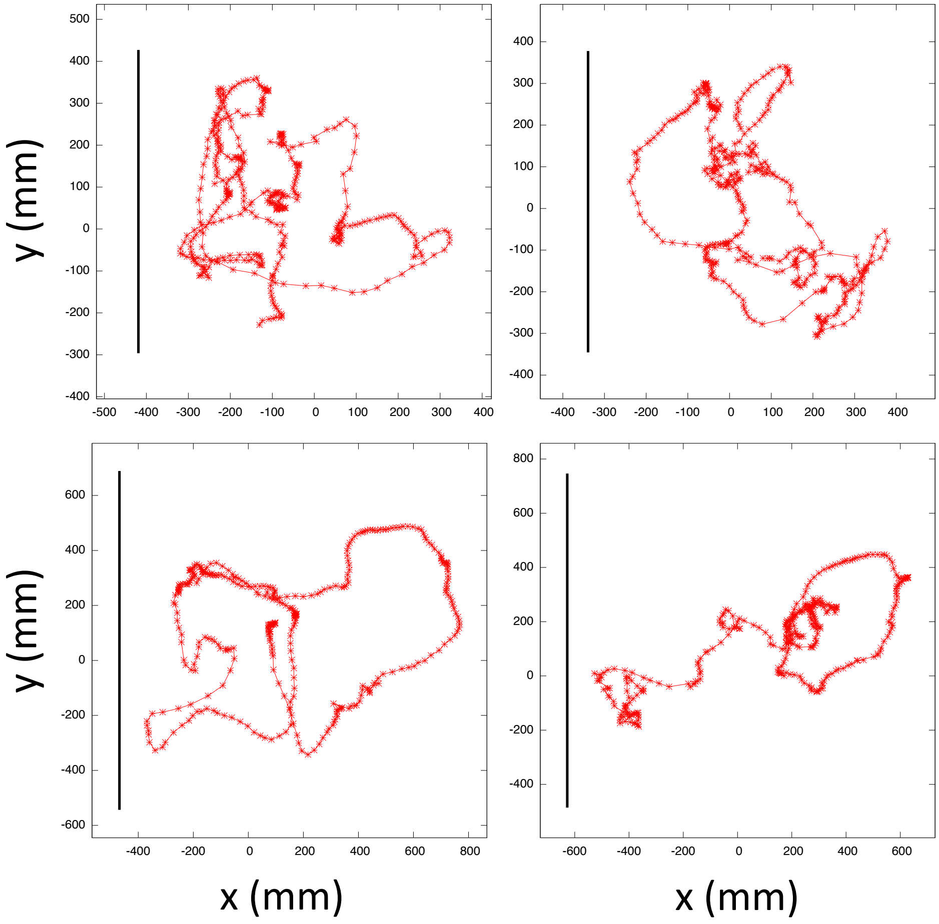

Figure S3. Trajectories of individual fish belonging to school with 20 individuals (above) and 30 individuals (below) in the center of reference frame. Scale bar represents school size, i.e., mean maximum distance between individuals shown in table 1. The axes are in millimeter.

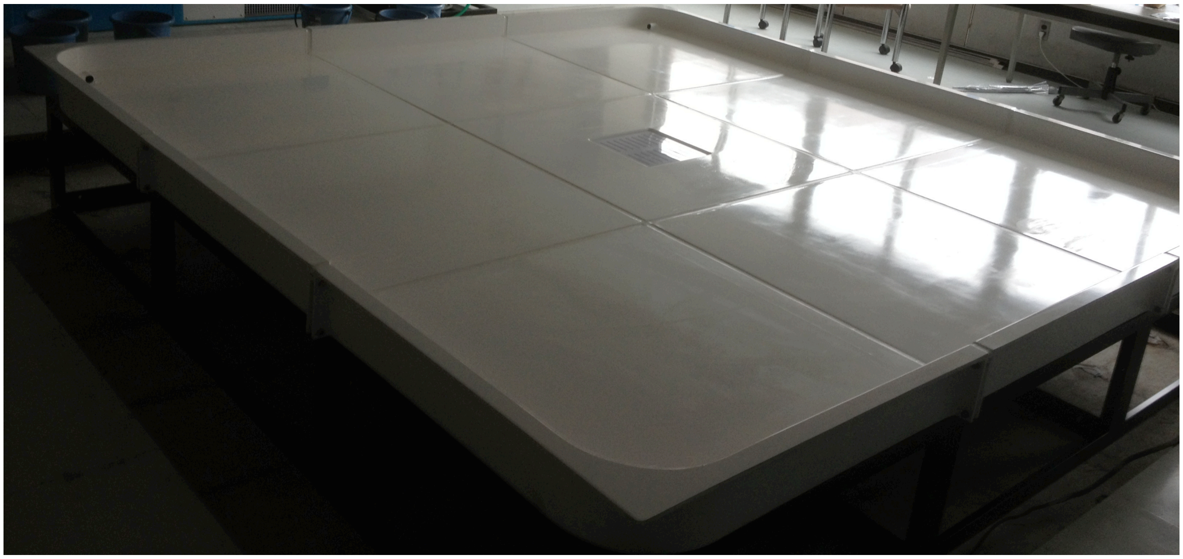
Figure S4. The experimental arena consisted of a 3 × 3 m white shallow tank.

		10 individuals		20 individuals		30 individuals		40 individuals	
		x_{min}=0.3	x_{min}=0.4	x_{min}=0.3	x_{min}=0.4	x_{min}=0.3	x_{min}=0.4	x_{min}=0.3	x_{min}=0.4
x_{max}		3.7		3.3		1.8		3.4	
N		336	267	204	158	101	82	75	60
Exponent	μ	2.00	2.13	2.12	2.26	2.28	2.99	2.20	2.52
	λ	1.94	1.82	2.24	2.11	3.29	3.66	2.27	2.24
wAIC	$w(tp)$	**1.00**	**0.99**	**0.99**	**0.99**	**0.99**	**0.99**	**0.99**	**0.99**
	$w(e)$	0.00	0.01	0.01	0.01	0.01	0.01	0.01	0.01
GOF	*P* value	**0.92**	**1.00**	**0.98**	**1.00**	**1.00**	**0.91**	**1.00**	**1.00**
	G statistic	299.9	120.6	162.7	85.8	36.3	65.0	17.2	15.1
Best fit model		Truncated power-law		Truncated power-law		Truncated power-law		Truncated power-law	

		50 individuals		60 individuals		40 individuals (milling)	
		x_{min}=0.3	x_{min}=0.4	x_{min}=0.3	x_{min}=0.4	x_{min}=0.3	x_{min}=0.4
x_{max}		4.1		3.5		3.8	
N		382	295	61	51	97	74
Exponent	μ	2.17	2.30	2.01	2.33	2.39	2.60
	λ	2.20	2.05	2.15	2.20	2.65	2.51
wAIC	$w(tp)$	**1.00**	**0.98**	0.47	**0.72**	**0.99**	**0.93**
	$w(e)$	0.00	0.02	**0.53**	0.28	0.01	0.07
GOF	*P* value	$<10^{-16}$	**0.48**	**1.00**	**1.00**	**0.99**	**1.00**
	G statistic	651.8	295.2	7.49	9.3	58.1	21.1
Best fit model		Neither		Neither		Truncated power-law	

Table S1. Data for distributions of contact duration, where N represents the total number of contact durations, and $w(tp)$ and $w(e)$ are AIC weights of truncated power-law and of exponential.

		***dr* = 15**	***dr* = 25**
N		436	261
x_{min}		29.5	29.9
x_{max}		1362.0	1161.4
Exponent	μ	1.80	1.94
	λ	0.009	0.011
wAIC	*w*(*tp*)	**1.00**	**1.00**
	w(*e*)	0.00	0.00
GOF	*P* value	**1.00**	**1.00**
	G statistic	47.1	89.6
Best fit model		Truncated power-law	Truncated power-law

Table S2. Data for distributions of step-lengths in the center of mass reference frame, displayed by 40 individuals school at *dt* = 15 and 25, where *N* represents the total number of step-lengths, and *w*(*tp*) and *w*(*e*) are AIC weights of truncated power-law and of exponential.

		10 individuals	20 individuals	30 individuals	40 individuals	50 individuals	60 individuals
N		519	332	630	401	751	242
x_{min}		27.0	25.7	21.9	24.6	26.2	25.7
x_{max}		532.3	506.1	971.5	1207.8	1112.7	849.2
Exponent	μ	2.33	1.88	1.88	1.86	2.07	1.91
	λ	0.024	0.015	0.014	0.011	0.015	0.014
wAIC	$w(tp)$	**1.00**	**1.00**	**1.00**	**1.00**	**1.00**	**1.00**
	$w(e)$	0.00	0.00	0.00	0.00	0.00	0.00
GOF	*P* value	**1.00**	**1.00**	**1.00**	**1.00**	**1.00**	**1.00**
	G statistic	206.5	77.1	360.7	108.1	503.5	105.5
Best fit model		Truncated power-law	Truncated power-law	Truncated power-law	Truncated power-law	Truncated power-law	Truncated power-law

Table S3. Data for distributions of step-lengths in the center of mass reference frame, displayed by each school, where *N* represents the total number of step-lengths, and $w(tp)$ and $w(e)$ are AIC weights of truncated power-law and of exponential.

		$i = 3$	$i = 4$	$i = 5$	$i = 6$	$i = 7$
N		51	53	50	50	55
x_{min}		28.6	29.8	25.6	27.7	29.7
x_{max}		255.9	215.1	256.9	532.3	286.0
Exponent	μ	2.04	2.08	2.18	2.31	1.99
	λ	0.024	0.026	0.027	0.019	0.021
wAIC	$w(tp)$	**0.96**	**0.94**	**0.98**	**1.00**	**1.00**
	$w(e)$	0.04	0.06	0.02	0.00	0.00
GOF	P value	**1.00**	**1.00**	**1.00**	**1.00**	**1.00**
	G statistic	4.24	3.97	4.90	12.3	4.82
Best fit model		Truncated power-law	Truncated power-law	Truncated power-law	Truncated power-law	Truncated power-law

Table S4. Data for distributions of step-lengths in the center of mass reference frame, displayed by each individual fish belonging to 10 individuals school, where i indicates individual number that corresponds as in figure S2, N represents the total number of step-lengths, and $w(tp)$ and $w(e)$ are AIC weights of truncated power-law and of exponential.
